# Supplementary material for: Transcutaneous Laryngeal Ultrasound for Vocal Cord Paralysis Assessment in Patients Undergoing Thyroid and Parathyroid Surgery—A Systematic Review and Meta-Analysis
Source: J Clin Med. 2021 Nov 19;10(22):5393. doi: 10.3390/jcm10225393 (PMC8617675; doi:10.3390/jcm10225393)

Table S1. Descriptions of various technical variations utilized during TLUSG examination.

Technical Aspect	Variations	Description
Approach	Transverse/Midline /Anterior	Probe is placed transversely below thyroid cartilage at the mid-level from anterior direction, angled superiorly, moved in craniocaudal direction until vocal cords are visualized.
	Lateral	Localize thyroid cartilage notch. Probe is placed on the lateral surface of the thyroid cartilage lamina, moved craniocaudally until vocal cords are visualized.
Maneuvers	Passive	Patient resting quietly. Ask patient to breath normally or slowly.
	Active	Asking the patient to phonate "a" vowel.
	Valsalva	Ask patient to perform valsalva maneuver, which leads to adduction of vocal cords to the midline. Vocal cords can be localized by moving the probe in cranio-caudal direction. Then, ask patient to breath normally to allow abduction of vocal cords in order to assess bilateral mobility.
Additional aspects	Gel Pad	Gel pad is placed on the neck skin of the patient with the USG probe positioned on the pad to allow tight contact and improve USG wave propagation.

Table S2. Patient selection criteria and study protocols for all included studies.

First Author, Publication Year, Country	Patient criteria	Patient Position	Study Protocol	VCP assessment
Borel et al. 2016, France	Exclusion: age under 18, suspected or confirmed extensive malignant disease and preoperative voice abnormalities	Supine, No neck elevation	DFL (POD 1, by an otolaryngologist)<br>→ TLUSG (POD1/2, by a radiologist, no TLUSG experience).	Landmark visualized: NR.<br><br>Diagnosis of VCP: VC immobility, decrease in mobility and/or asymmetry
de Miguel et al. 2017, Spain	Exclusion: urgent surgery, a history of intolerance to the VL procedure, and having undergone surgical procedures in which the thyroid was not completely removed.	Supine decubitus, slightly extended neck	Preop Period:<br>VL (day before surgery by an experienced phoniatrics specialists)<br>→TLUSG (operation day, by anesthesiologist with experience of 40 TLUSG exams).<br><br>Postop Period:<br>TLUSG (after pain and emesis controlled, by same anesthesiologist)<br>→ VL (POD 4, by an experienced phoniatrics specialists)	Landmarks visualized: TVC, FVC, AF.<br><br>Diagnosis of VCP: Decreased or absent movement of one or both TVC or AF
Gambardella et al. 2020, Italy	Exclusion: age < 16 years, previous neck surgery and irradiation, presence of tracheostomy, pre-existing diagnosis of vocal cord nodules.	Supine, Extended Neck	Preoperative TLUS (by an experienced examiner)<br>→ DFL (by an otolaryngologist).	Landmarks visualized: TVC, FVC, AF.<br><br>Diagnosis of VCP: Weakness, asymmetry and paralysis of VC
Kandil et al. 2016, USA	NR	Supine, Extended Neck	TLUSG (by operating surgeon)<br>→DFL (by operating surgeon) (8 years' experience).<br>Performed preoperatively and on POD 7.	Landmarks visualized: TVC, FVC, AF.<br><br>Diagnosis of VCP: Weakness, asymmetry and paralysis of VC
Kilic et al. 2017, Turkey	Exclusion: <18 years old, previous neck surgery, and having previous VCP	Supine, Extended Neck	TLUSG (by sonographers), DFL (otolaryngologists) (order not described).	Landmarks visualized: NR. B-mode US was used to localize vibrating VC. Pulsed Doppler mode to determine the range of tissue motion velocity.

			Performed preoperatively (1–3 days before surgery) and on POD 7–10.	Diagnosis of VCP: Grade II and III.
Knyazeva et al. 2018, Germany	Exclusion: patients in which preoperative DFL was not possible due to technical and/or anatomical reasons	Supine, slightly extended neck	TLUSG → DFL<br>(By experienced surgeons in neck USG and DFL (>500/year), experience with TLUSG not mentioned).<br>Performed preoperatively (day of surgery) and on POD 1.	Landmarks visualized: NR. B-mode US was used to localize vibrating VC. Pulsed Doppler mode to determine the range of tissue motion velocity.<br><br>Diagnosis of VCP: Grade II and III.
Rybakovas et al. 2019, Lithuania	Exclusion: <18 years old, pregnancy, refusal to participate in the study, and presence of RLN palsy before the surgery	NA	Preoperative TLUSG (by surgeon, trained at a single course)<br>→ DFL (on POD1)	Landmarks visualized: TVC, FVC, AF. Intraoperative TLUSG examination was defined as assessable if ≥1 landmark were identified.<br><br>Diagnosis of VCP: Observed movement in any of 3 landmarks was considered as sufficient proof of normal RLN function.
Shah et al. 2019, India	Exclusion: Patients with suspected or confirmed extensive malignant disease, preoperative voice abnormalities, Mallampatti grade III and IV, patients with limited neck extension, previous neck surgeries and patient's refusing to participate in the study.	NA	VL + TLUSG (immediately after surgery, performed by two separate anesthesiologist)	NR
Wong et al. 2013, Hong Kong	NR	Supine, slightly extended neck, Arms on side	TLUSG (by endocrine surgeon) → DFL (by experienced endoscopist).<br>Performed preoperatively (1 day before) and on POD 7–10.	Landmarks visualized: TVC, FVC.<br><br>Diagnosis of VCP: Grade II and III.
Wong et al. 2015, Hong Kong	NR	Supine, slightly extended neck, Arms on side	TLUSG (by endocrine surgeon) → DFL (by experienced endoscopist).<br>Performed preoperatively (1 day before) and on POD 7.	Landmarks visualized: TVC, FVC. TLUSG examination was defined as assessable if ≥1 landmark were identified.<br><br>Diagnosis of VCP: Any reduced or absent movement in >1 VC on TLUSG or DL

Wong et al. 2019, Hong Kong	NR	Supine, slightly extended neck, Arms on side	Evaluation of Voice complaints, VHI-30 Q.<br>TLUSG (by endocrine surgeon) → DFL (by experienced endoscopist).<br>Performed preoperatively (1–7 days before) and on POD 7–10.	Landmarks visualized: TVC, FVC, AF. TLUSG examination was defined as assessable if ≥1 landmark were identified.<br><br>Diagnosis of VCP: Grade II and III
Wong et al. 2016, Hong Kong	NR	Supine, slightly extended neck, Arms on side	TLUSG (by operating surgeons, experience of >200 TLUSG exams) → DFL (by endoscopist).<br>Performed preoperatively (1 day before) and on POD 7–10.	Landmarks visualized: TVC, FVC, AF. TLUSG examination was defined as assessable if ≥1 landmarks of both VCs were identified.<br><br>Diagnosis of VCP: Any reduced or absent movement in >1 sonographic landmark of VCs on TLUSG or DL on one maneuver
Woo et al. 2016, Seoul	NR	Supine, slightly extended neck	TLUSG -> DFL (different accessors).<br>Performed preoperative and postoperatively	Landmarks visualized: TVC, FVC, AF. Identifying all three sonographic landmarks was not mandatory. TLUSG examination was defined as assessable if ≥1 landmark were identified.<br><br>Diagnosis of VCP: visualizing normal movement in one of the LUS landmarks was sufficient to exclude VCP. Grade II and III. After conventional laryngeal US, a gel pad was placed on the patient's neck skin, and an US transducer was positioned on the gel pad to ensure tight contact and to enhance US penetration.
Woo et al. 2016, Seoul	NR	Supine, slightly extended neck	TLUSG -> DFL (different accessors).<br>Performed preoperative and postoperatively	Landmarks visualized: TVC, FVC, AF.<br><br>Diagnosis of VCP: Grade II and III
Woo et al. 2016, Seoul	NR	NR	TLUSG (by 1 endocrine surg) -> DLF (by 3 endocrine surg).<br>Performed preoperative and on POD 10–14.	Landmarks visualized: TVC, FVC, AF. TLUSG examination was defined as assessable if ≥1 landmark were identified.<br><br>Diagnosis of VCP: Grade II and III

Wong et al. 2014, Hong Kong	Exclusion: VCP in preoperative period	Supine, slightly extended neck, Arms on side	TLUSG (by operating surgeons, minimal experience (half-day training)) → DFL (by endoscopist).<br>Performed preoperatively (1 day before) and on POD 7–10.	Landmarks visualized: TVC, FVC, AF. TLUSG examination was defined as assessable if ≥1 landmark were identified.<br><br>Diagnosis of VCP: decreased or no movement in any of the 3 landmarks

(NR = not reported, VCP = vocal cord paralysis, DFL = direct flexible laryngoscopy, VL = videostrobolaryngoscopy, RLN = recurrent laryngeal nerve, POD = postoperative day, TLUSG = transcutaneous laryngeal ultrasonography, USG = ultrasonography, VHI-30 Q = voice handicap index-30 questionnaire, TVC = true vocal cord, FVC = false vocal cord, AF = arytenoid folds, VCP = vocal cord paralysis rate, PPV = Positive predictive value, NPV = negative predictive value).

Table S3. Summary of diagnostic accuracy statistics and summary estimates for studies analyzing TLUSG in preoperative period. 95% Confidence intervals for summary estimates are reported in brackets, where applicable.

Preoperative TLUSG	**Author**	**No. of Patients#**	**TP**	**FP**	**FN**	**TN**	**VR%**	**VCP%**	**Sensitivity**	**Specificity**	**PPV**	**NPV**
All studies	de Miguel et al. 2017	93	2	0	1	90	94.00	3.20	0.667 (0.119-0.983)	1.0 (0.959-1.0)	1.0 (0.177-1.0)	0.989 (0.94-0.999)
	Gambardella et al. 2020	396	NR	NR	NR	NR	96.50	7.80	0.969 (0.944-0.982)	0.956 (0.93-0.973)	0.652 (0.603-0.699)	0.997 (0.983-1.0)
	Knyazeva et al. 2018	526	2	0	1	523	79.00	0.45	0.667 (0.119-0.983)	1.0 (0.994-1.0)	1.0 (0.178-1.0)	0.999 (0.992-0.999)
	**Summary**	**1015**					**86.28**	**3.25**	**0.785 (NC)^**	**0.983 (NC)^**	**0.864 (NC)^**	**0.997 (NC)^**
Transverse + Lateral	de Miguel et al. 2017	93	2	0	1	90	94.00	3.20	0.667 (0.119-0.983)	1.0 (0.959-1.0)	1.0 (0.177-1.0)	0.989 (0.94-0.999)
	Gambardella et al. 2020	396	NR	NR	NR	NR	96.46	7.80	0.969 (0.944-0.982)	0.956 (0.93-0.973)	0.652 (0.603-0.699)	0.997 (0.983-1.0)
	**Summary**	**489**					**95.93**	**6.93**	**0.912 (NC)^**	**0.964 (NC)^**	**0.718 (NC)^**	**0.996 (NC)^**
Transverse alone	Knyazeva et al. 2018	526	2	0	1	523	79.00	0.45	0.667 (0.119-0.983)	1.0 (0.994-1.0)	1.0 (0.178-1.0)	0.999 (0.992-0.999)
	**Summary**	**526**					**79.00**	**0.45**	**0.667 (0.119-0.983)**	**1.0 (0.994-1.0)**	**1.0 (0.178-1.0)**	**0.999 (0.992-0.999)**
Valsalva Maneuver	Gambardella et al. 2020	396	NR	NR	NR	NR	96.46	7.80	0.969 (0.944-0.982)	0.956 (0.93-0.973)	0.652 (0.603-0.699)	0.997 (0.983-1.0)
	Knyazeva et al. 2018	526	2	0	1	523	79.00	0.45	0.667 (0.119-0.983)	1.0 (0.994-1.0)	1.0 (0.178-1.0)	0.999 (0.992-0.999)
	**Summary**	**922**					**85.50**	**3.61**	**0.797 (NC)^**	**0.981 (NC)^**	**0.851 (NC)^**	**0.998 (NC)^**
No Valsalva Maneuver	de Miguel et al. 2017	93	2	0	1	90	94.00	3.20	0.667 (0.119-0.983)	1.0 (0.959-1.0)	1.0 (0.177-1.0)	0.989 (0.94-0.999)
	**Summary**	**93**					**94.00**	**3.20**	**0.667 (0.119-0.983)**	**1.0 (0.959-1.0)**	**1.0 (0.177-1.0)**	**0.989 (0.94-0.999)**

(TLUSG = transcutaneous laryngeal ultrasonography, TP = true positive, FP = false positive, FN = false negative, TN = true negative, VR = vocal cord visualization rate, VCP = vocal cord paralysis rate, PPV = Positive predictive value, NPV = negative predictive value, NR = not reported, NC = not calculated). # Patients in whom vocal cords were visualized were considered for meta-analysis of diagnostic accuracy. ^ Calculation of 95% confidence interval was not possible due to lack of data in one of the studies in the given analyses.

Table S4. Summary of diagnostic accuracy statistics and summary estimates for studies analyzing TLUSG in postoperative period. 95% Confidence intervals for summary estimates are reported in brackets, where applicable.

Postoperative TLUSG	**Author**	**No. of Patients#**	**TP**	**FP**	**FN**	**TN**	**VR%**	**VCP%**	**Sensitivity**	**Specificity**	**PPV**	**NPV**
All studies	Borel et al. 2016	95	3	4	6	82	72.80	9.50	0.333 (0.121-0.649)	0.954 (0.886-0.982)	0.429 (0.158-0.75)	0.932 (0.859-0.968)
	de Miguel et al. 2017	93	14	3	1	75	93.00	16.10	0.933 (0.702-0.997)	0.962 (0.893-0.989)	0.824 (0.589-0.938)	0.987 (0.929-0.999)
	Kilic et al. 2017	314	21	2	4	287	96.60	7.90	0.84 (0.654-0.936)	0.993 (0.975-0.999)	0.913 (0.732-0.985)	0.986 (0.965-0.995)
	Knyazeva et al. 2018	526	34	6	4	482	NR	7.20	0.895 (0.759-0.958)	0.987 (0.973-0.994)	0.85 (0.709-0.929)	0.992 (0.979-0.997)
	Rybakovas et al. 2019	112	5	3	1	103	100.00	5.40	0.833 (0.437-0.992)	0.972 (0.92-0.992)	0.625 (0.306-0.863)	0.99 (0.948-0.999)
	Shah et al. 2019	45	3	2	1	39	100.00	8.89	0.75 (0.30-0.987)	0.951 (0.839-0.991)	0.6 (0.231-0.928)	0.975 (0.871-0.999)
	Wong et al. 2019	1132	58	63	10	1001	94.60	6.01	0.853 (0.75-0.918)	0.941 (0.925-0.953)	0.479 (0.392-0.568)	0.99 (0.982-0.995)
	**Summary Estimate**	**2317**					**94.13**	**7.11**	**0.839 (0.772-0.885)**	**0.962 (0.952-0.969)**	**0.643 (0.559-0.686)**	**0.987 (0.981-0.991)**
Transverse + Lateral/Gel Pad	de Miguel et al. 2017	93	14	3	1	75	93.00	16.10	0.933 (0.702-0.997)	0.962 (0.893-0.989)	0.824 (0.589-0.938)	0.987 (0.929-0.999)
	Kilic et al. 2017	314	21	2	4	287	96.60	7.90	0.84 (0.654-0.936)	0.993 (0.975-0.999)	0.913 (0.732-0.985)	0.986 (0.965-0.995)
	Rybakovas et al. 2019	112	5	3	1	103	100.00	5.40	0.833 (0.437-0.992)	0.972 (0.92-0.992)	0.625 (0.306-0.863)	0.99 (0.948-0.999)
	Wong et al. 2013	193	14	4	1	174	94.60	7.77	0.933 (0.702-0.997)	0.978 (0.944-0.991)	0.779 (0.548-0.91)	0.994 (0.968-0.999)
	**Summary Estimate**	**712**					**96.04**	**8.54**	**0.876 (0.782-0.943)**	**0.982 (0.968-0.989)**	**0.819 (0.709-0.893)**	**0.989 (0.978-0.995)**
Transverse alone	Borel et al. 2016	95	3	4	6	82	72.80	9.50	0.333 (0.121-0.649)	0.954 (0.886-0.982)	0.429 (0.158-0.75)	0.932 (0.859-0.968)
	Knyazeva et al. 2018	526	34	6	4	482	NR	7.20	0.895 (0.759-0.958)	0.987 (0.973-0.994)	0.85 (0.709-0.929)	0.992 (0.979-0.997)
	Shah et al. 2019	45	3	2	1	39	100.00	8.89	0.75 (0.30-0.987)	0.951 (0.839-0.991)	0.6 (0.231-0.928)	0.975 (0.871-0.999)
	Wong et al. 2019	1132	58	63	10	1001	94.60	6.01	0.853 (0.75-0.918)	0.941 (0.925-0.953)	0.479 (0.392-0.568)	0.99 (0.982-0.995)
	**Summary Estimate**	**1798**					**93.11**	**6.61**	**0.835 (0.745-0.882)**	**0.955 (0.944-0.964)**	**0.588 (0.492-0.638)**	**0.987 (0.980-0.992)**
Valsalva Maneuver	Knyazeva et al. 2018	526	34	6	4	482	NR	7.20	0.895 (0.759-0.958)	0.987 (0.973-0.994)	0.85 (0.709-0.929)	0.992 (0.979-0.997)
	Wong et al. 2019	1132	58	63	10	1001	94.60	6.01	0.853 (0.75-0.918)	0.941 (0.925-0.953)	0.479 (0.392-0.568)	0.99 (0.982-0.995)
	**Summary Estimate**	**1658**					**NA***	**6.39**	**0.866 (0.790-0.919)**	**0.956 (0.944-0.965)**	**0.597 (0.494-0.645)**	**0.991 (0.984-0.994)**
No Valsalva Maneuver	Borel et al. 2016	95	3	4	6	82	72.80	9.50	0.333 (0.121-0.649)	0.954 (0.886-0.982)	0.429 (0.158-0.75)	0.932 (0.859-0.968)
	de Miguel et al. 2017	93	14	3	1	75	93.00	16.10	0.933 (0.702-0.997)	0.962 (0.893-0.989)	0.824 (0.589-0.938)	0.987 (0.929-0.999)
	Kilic et al. 2017	314	21	2	4	287	96.60	7.90	0.84 (0.654-0.936)	0.993 (0.975-0.999)	0.913 (0.732-0.985)	0.986 (0.965-0.995)
	Wong et al. 2015	552	40	25	4	483	95.00	7.97	0.909 (0.788-0.964)	0.951 (0.928-0.966)	0.615 (0.494-0.724)	0.992 (0.979-0.997)

	**Summary Estimate**	**1054**					**93.23**	**8.80**	**0.839 (0.751-0.899)**	**0.965 (0.951-0.975)**	**0.705 (0.606-0.774)**	**0.984 (0.974-0.990)**

(TLUSG = transcutaneous laryngeal ultrasonography, TP = true positive, FP = false positive, FN = false negative, TN = true negative, VR = vocal cord visualization rate, VCP = vocal cord paralysis rate, PPV = Positive predictive value, NPV = negative predictive value, NR = not reported). # Patients in whom vocal cords were visualized were considered for meta-analysis of diagnostic accuracy. * Analysis was not possible since one of the studies in the respective analysis did not provide data on visualization rate in their cohort.

Table S5. Summary of diagnostic accuracy statistics and summary estimates for subgroup analysis based on the specialty of the TLUSG examiner. 95% Confidence intervals for summary estimates are reported in brackets.

Postoperative Period	Author	Experience	N of Patients	TP	FP	FN	TN	VR	VCP rate	Sn	Sp	PPV	NPV
**Radiologist**	Borel et al. 2016	No experience	95	3	4	6	82	72.80	9.50	0.333 (0.121-0.649)	0.954 (0.886-0.982)	0.429 (0.158-0.75)	0.932 (0.859-0.968)
	Kilic et al. 2017	NR	314	21	2	4	287	96.60	7.90	0.84 (0.654-0.936)	0.993 (0.975-0.999)	0.913 (0.732-0.985)	0.986 (0.965-0.995)
	**Summary Estimate**		**409**					**90.95**	**8.27**	**0.722 (0.538-0.832)**	**0.984 (0.966-0.993)**	**0.801 (0.627-0.905)**	**0.974 (0.952-0.986)**
**Anesthesiologist**	de Miguel et al. 2017	40 examinations	93	14	3	1	75	93.00	16.10	0.933 (0.702-0.997)	0.962 (0.893-0.989)	0.824 (0.589-0.938)	0.987 (0.929-0.999)
	Shah et al. 2019	NR	45	3	2	1	39	100.00	8.89	0.75 (0.30-0.987)	0.951 (0.839-0.991)	0.6 (0.231-0.928)	0.975 (0.871-0.999)
	**Summary Estimate**		**138**					**95.06**	**8.2**	**0.873 (0.686-0.981)**	**0.958 (0.905-0.982)**	**0.751 (0.566-0.899)**	**0.983 (0.939-0.997)**
**Radiologist + Anesthesiologist**	Borel et al. 2016	No experience	95	3	4	6	82	72.80	9.50	0.333 (0.121-0.649)	0.954 (0.886-0.982)	0.429 (0.158-0.75)	0.932 (0.859-0.968)
	Kilic et al. 2017	NR	314	21	2	4	287	96.60	7.90	0.84 (0.654-0.936)	0.993 (0.975-0.999)	0.913 (0.732-0.985)	0.986 (0.965-0.995)
	de Miguel et al. 2017	40 examinations	93	14	3	1	75	93.00	16.10	0.933 (0.702-0.997)	0.962 (0.893-0.989)	0.824 (0.589-0.938)	0.987 (0.929-0.999)
	Shah et al. 2019	NR	45	3	2	1	39	100.00	8.89	0.75 (0.30-0.987)	0.951 (0.839-0.991)	0.6 (0.231-0.928)	0.975 (0.871-0.999)
	**Summary Estimate**		**547**					**92.03**	**7.00**	**0.759 (0.645-0.866)**	**0.978 (0.961-0.988)**	**0.788 (0.659-0.878)**	**0.976 (0.958-0.986)**
**Operating Surgeon**	Knyazeva et al. 2018	NR	526	34	6	4	482	**NR**	7.20	0.895 (0.759-0.958)	0.987 (0.973-0.994)	0.85 (0.709-0.929)	0.992 (0.979-0.997)
	Rybakovas et al. 2019	Single training course	112	5	3	1	103	100.00	5.40	0.833 (0.437-0.992)	0.972 (0.92-0.992)	0.625 (0.306-0.863)	0.99 (0.948-0.999)
	Wong et al. 2019	>500 TLUSG/year	1132	58	63	10	1001	94.60	6.01	0.853 (0.75-0.918)	0.941 (0.925-0.953)	0.479 (0.392-0.568)	0.99 (0.982-0.995)
	**Summary Estimate**		**1770**					**95.06**	**6.33**	**0.864 (0.791-0.917)**	**0.957 (0.946-0.965)**	**0.599 (0.499-0.646)**	**0.991 (0.985-0.994)**

(TLUSG = transcutaneous laryngeal ultrasonography, TP = true positive, FP = false positive, FN = false negative, TN = true negative, VR = vocal cord visualization rate, VCP = vocal cord paralysis rate, PPV = Positive predictive value, NPV = negative predictive value, NR = not reported).

Table S6. Characteristics of studies assessing technical aspects of TLUSG examination.

**Study Characteristics**				**TLUSG Examination**			**Cohort Demographics**		
First Author, Publication Year, Country	Accrual years	Study Design, Blinding	Reference Method	USG probe, frequency	TLUSG Approach	Maneuver used	No. of patients	Female%	Mean age
Wong et al. 2016, Hong Kong	NR	P, Y	DFL	Linear, 5-10 MHz	Transverse Approach	PAV	342	78.1	51[#]
Woo et al. 2016, Seoul	NR	P, Y	DFL	Linear, 3-9 MHz	Transverse Approach → gel pad	PAV	482	79.3	48[#]
Woo et al. 2016, Seoul	NR	P, Y	DFL	Linear, 5-12 MHz OR 3-9 MHz	Transverse Approach	PAV	301	82.7	48[#]
Woo et al. 2016, Seoul	NR	P, Y	DFL	Linear, 3-12 MHz	Transverse for females, Lateral for males	PV	382	78.5	48
Wong et al. 2014	2013	P, Y	DFL	Linear, 5-10 MHz OR 3-12 MHz	Transvers Approach	PA	245	81.2	50.5[#]

(NR = not reported, P = Prospective study, Y = yes, DFL = direct flexible laryngoscopy, TLUSG = transcutaneous laryngeal ultrasonography, A = active phonation, P=passive breathing, V = valsalva maneuver). [#] Median age.

Table S7. Summary of visualization rates and diagnostic accuracy of studies assessing technical aspects of TLUSG examination

First Author, Publication Year, Country	Grouping	N. of patients	TP	FP	FN	TN	VR%	VCP%	Sensitivity%	Specificity%	PPV%	NPV%
Wong et al. 2016, Hong Kong	Passive	342	16	17	1	279	91.50	3.3	94.1 (73-99.7)	94.3 (91-96.4)	48.5 (32.5-65.8)	99.6 (98-99)
	Active		12	7	4	284	89.80		75 (50.5-89.8)	97.6 (95.1-98.8)	63.2 (41-80.9)	98.6 (96.5-99)
	**Valsalva**		13	8	3	291	**92.10**		81.3 (56.9-93.4)	97.3 (94.8-98.6)	61.9 (40.8-79.3)	98.9 (97-99)
Woo et al. 2016, Seoul	TLUSG	482	50	3	1	396	93.40	10.6	98 (89.7-99)	99.3 (97.8-99.8)	94.3 (84.6-98.5)	99.8 (98.6-99)
	**TLUSG-Gel Pad**		50	3	1	423	**99.00**		98 (89.7-99)	99.3 (97.9-99.8)	94.3 (84.6-98.5)	99.8 (98.6-99)
Woo et al. 2016, Seoul	High Frequency	301	39	2	1	224	88.40	14	97.5 (87.1-99.9)	99.1 (96.8-99.8)	95.1 (83.9-99.1)	99.6 (97.5-99)
	**Low Frequency**		41	2	1	250	**97.70**		97.6 (87.7-99.9)	99.2 (97.2-99.9)	95.4 (84.5-99.2)	99.6 (97.9-99)
Woo et al. 2016, Seoul	Overall	382	23	3	0	356	100.00	6	100 (85.7-100)	99.2 (97.6-99.8)	88.5 (71-96)	100 (98.9-100)
	Transverse Approach		18	2	0	280	100.00		100 (82.4-100)	99.3 (97.5-99)	90 (69-98.2)	100 (98.7-100)
	Lateral Approach		5	1	0	76	100.00		100 (56.6-100)	98.7 (93-99)	83.3 (43.7-99.2)	100 (95.2-100)
Wong et al. 2014, Hong Kong	Visualizing any 1 landmark	245	2	0	0	16	FVC = 92.7<br>TVC = 36.7<br>AF = 89.8	4.3	100 (17.8-100)	100 (80.6-100)	100 (17.8-100)	100 (80.6-100)
	Visualizing any 2 landmarks		9	10	0	107	FVC+TVC = 36.3<br>FVC+AF = 87.3<br>TVC+AF = 36.7		100 (70.1-100)	91.5 (84.9-95.3)	47.4 (27.3-68.3)	100 (96.5-100)
	Visualizing all 3 landmarks		6	9	0	74	FVC+TVC+AF = 36.3		100 (60.9-100)	89.2 (80.7-94.2)	40 (19.8-64.3)	100 (95.1-100)

(TLUSG = transcutaneous laryngeal ultrasonography, TP = true positive, FP = false positive, FN = false negative, TN = true negative VR = vocal cord visualization rate, TVC = true vocal cord, FVC = false vocal cord, AF = arytenoid folds, VCP = vocal cord paralysis rate, PPV = Positive predictive value, NPV = negative predictive value)

Table S8. QUADAS-2 risk of bias assessment of studies included in the quantitative and qualitative analysis.

Study	RISK OF BIAS				APPLICABILITY CONCERNS		
	PATIENT SELECTION	INDEX TEST	REFERENCE STANDARD	FLOW AND TIMING	PATIENT SELECTION	INDEX TEST	REFERENCE STANDARD
Borel et al. 2016, France	☺	☺	☺	☹	☺	☺	☺
de Miguel et al. 2017, Spain	☹	☺	☺	☹	☺	☺	☺
Gambardella et al. 2020, Italy	☺	☺	☺	☺	☺	☺	☺
Kandil et al. 2016, USA	?	☹	☹	☺	☺	☹	☺
Kilic et al. 2017, Turkey	☺	?	?	☹	☺	☹	☺
Knyazeva et al. 2018, Germany	☺	☺	☺	☺	☺	☺	☺
Rybakovas et al. 2019, Lithuania	☺	?	?	☺	☺	☹	☺
Shah et al. 2019, India	☺	☺	☺	☺	☺	☺	☺
Wong et al. 2013, Hong Kong	☺	☺	☺	☹	☺	☺	☺
Wong et al. 2015, Hong Kong	☺	☺	☺	☹	☺	☺	☺
Wong et al. 2019, Hong Kong	☺	☺	☺	☹	☺	☺	☺
Wong et al. 2016, Hong Kong	☺	☺	☺	☹	☺	☺	☺
Woo et al. 2016, Seoul	☺	☺	☺	☺	☹	☺	☺
Woo et al. 2016, Seoul	☺	☺	☺	☺	☹	☺	☺
Woo et al. 2016, Seoul	☺	☺	☺	☺	☹	☺	☺
Wong et al. 2014, Hong Kong	☺	☺	☺	☹	☺	☺	☺

☺ Low Risk ☹ High Risk ? Unclear Risk

Figure S1. PRISMA Flowchart.

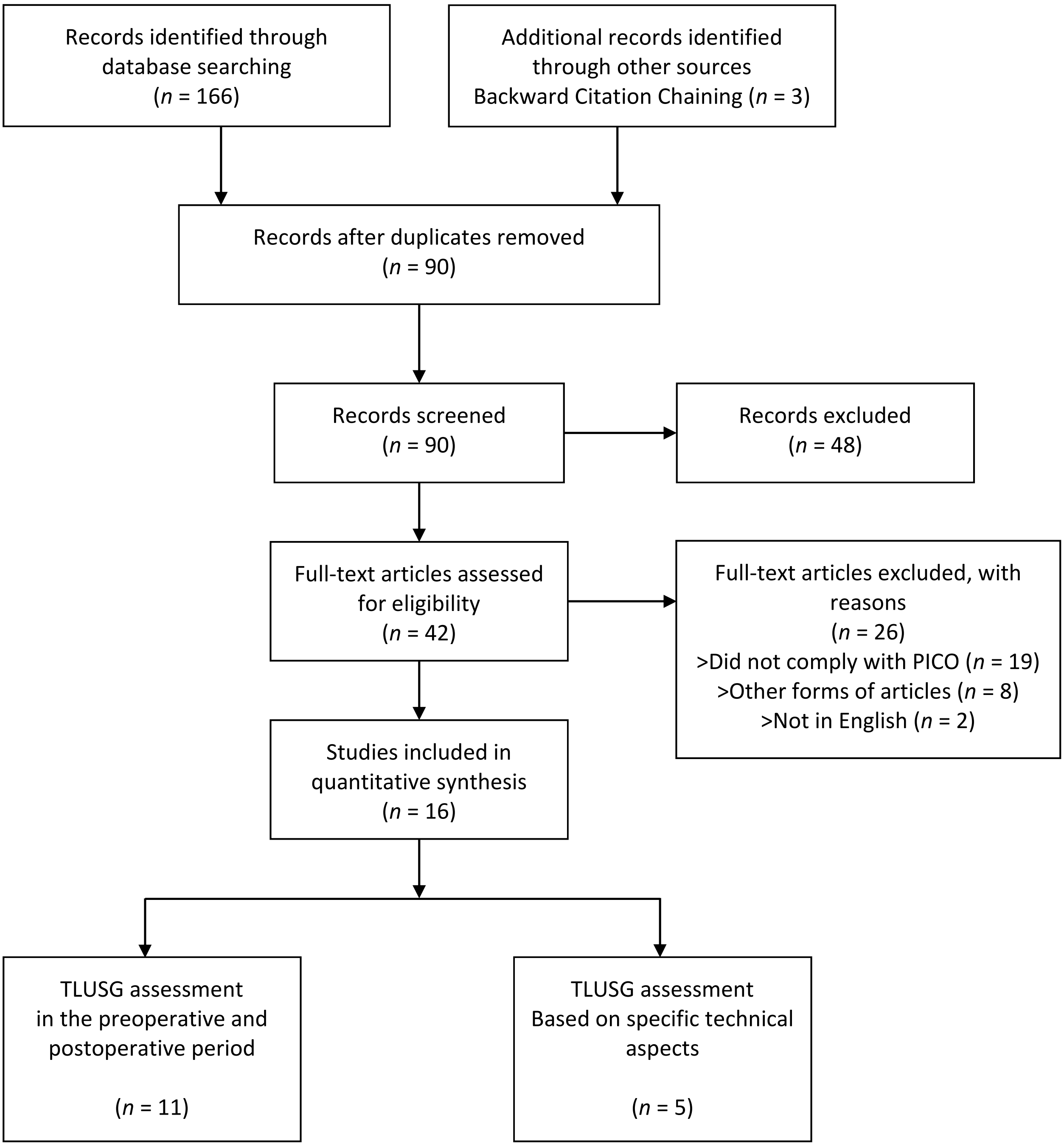

Supplement: Supplementary file 1 [file jcm-10-05393-s001.zip › jcm-1459737-supplementary.pdf]
